# Cardiovascular Diseases in the Critically Ill: Bridging Evidence Gaps Between Cardiology and Intensive Care Medicine

**DOI:** 10.3390/biomedicines14071530

**Published:** 2026-07-08

**Authors:** Vasileios Vazgiourakis

**Affiliations:** 1 Faculty of Medicine, School of Health Sciences, University of Thessaly, 3 Panepistimiou Street, Biopolis, 41500 Larissa, Greece; vasvazg@yahoo.com; 2 Department of Critical Care, University Hospital of Larissa, Mezourlo, 41110 Larissa, Greece

## 1. Introduction

Cardiovascular disease remains a leading contributor to global mortality and morbidity [1]; however, its diagnosis and treatment become particularly complex when cardiovascular dysfunction develops during sepsis, shock, respiratory failure, postoperative deterioration, or extracorporeal support [2,3]. In the intensive care unit (ICU), myocardial injury, arrhythmias, ventricular dysfunction, thrombosis, and bleeding often coexist, creating a cardiovascular phenotype that differs substantially from conventional cardiology practice. Cardiovascular disease in critical illness should therefore not be considered a single-organ problem. It is a dynamic syndrome shaped by inflammation, altered loading conditions, hypoxemia, vasopressor exposure, renal and hepatic dysfunction, mechanical ventilation, frailty, anticoagulation complexity, and the availability of advanced circulatory support. This Special Issue, “The Treatment of Cardiovascular Diseases in the Critically Ill”, brings together contributions that reflect this evolving interface between cardiology and intensive care medicine, where diagnosis, prognosis, organ support, and survivorship must be considered together.

## 2. Diagnostic Ambiguity in the ICU

A central challenge is diagnostic ambiguity. Elevated cardiac troponin in critically ill patients should prompt structured phenotyping rather than automatic labeling as acute coronary syndrome. The Fourth Universal Definition of Myocardial Infarction is helpful because it distinguishes myocardial injury from infarction and separates type 1 myocardial infarction from type 2 [4]. Nevertheless, ICU patients often have simultaneous triggers, including hypoxemia, anemia, tachyarrhythmia, renal failure, vasopressors, systemic inflammation, and supply–demand mismatch. Contemporary acute coronary syndrome guidelines provide essential standards of care, but the sickest ICU patients are often underrepresented in the trials that inform these recommendations [5].

Sepsis-induced cardiomyopathy illustrates this uncertainty particularly well. Left ventricular ejection fraction is load-dependent, and septic vasoplegia may make “normal” or “reduced” ejection fraction misleading. Recent work emphasizes serial echocardiography, tissue Doppler, global longitudinal strain, biomarkers, and hemodynamic context, but universally accepted diagnostic criteria and targeted treatment algorithms remain lacking [6,7]. Similarly, Takotsubo syndrome, myocarditis, right ventricular failure, and non-ischemic myocardial injury may overlap clinically with acute coronary syndromes and cardiogenic shock, requiring careful integration of ECG findings, biomarkers, imaging, and clinical context [8,9].

## 3. Mechanical Circulatory Support: Promise, Limits, and Patient Selection

Mechanical circulatory support has become an increasingly important component of cardiovascular critical care, but it is not automatically beneficial. Venoarterial extracorporeal membrane oxygenation (VA-ECMO), extracorporeal cardiopulmonary resuscitation (ECPR), ventricular unloading devices, and temporary right ventricular support can provide lifesaving stabilization. Recent randomized evidence has also renewed interest in microaxial flow pump support for selected patients with ST-segment elevation myocardial infarction-related cardiogenic shock, while reinforcing the need to balance hemodynamic benefit against device-related complications [10]. Their benefit, however, depends on careful patient selection, timely initiation, unloading strategy, anticoagulation, weaning, neurological prognosis, and destination planning. Standardized shock staging frameworks may help align inclusion criteria, escalation thresholds, and outcome reporting across studies [11]. The ECLS-SHOCK trial did not show a mortality benefit from routine early VA-ECMO in infarct-related cardiogenic shock [12]. ECPR studies have also produced heterogeneous findings, with benefit for highly selected refractory ventricular fibrillation in ARREST [13] and neutral results in broader refractory out-of-hospital cardiac arrest populations in INCEPTION [14]. These findings suggest that the future of mechanical circulatory support will be defined less by broader use and more by precision selection and protocolized multidisciplinary care.

## 4. Contributions to This Special Issue

The articles collected in this Special Issue address several clinically important gaps. One theme is the need for better prognostication in patients with advanced heart failure and critical illness. Contributions focused on advanced heart failure risk stratification and frailty emphasize that prognosis is not determined solely by cardiac function. Frailty, comorbidity burden, sodium levels, blood pressure, coronary disease, prior healthcare utilization, and functional reserve should be incorporated into risk assessment. This perspective aligns with contemporary heart failure guidance and with growing evidence that frailty is common in heart failure and strongly associated with adverse outcomes [15,16].

A second theme is the transition from rescue therapy toward structured mechanical support pathways. Contributions addressing fulminant myocarditis supported with VA-ECMO, post-heart transplant right ventricular primary graft dysfunction treated with Impella RP Flex, and levosimendan use after ECPR highlight several unresolved questions: when to initiate support, how to identify reversibility, how to facilitate myocardial recovery, and how to define bridge-to-recovery, bridge-to-decision, or bridge-to-transplant strategies. These issues remain clinically relevant as pharmacological facilitation of myocardial recovery and ECMO weaning continues to be investigated [17].

A third theme is the recognition that complications of advanced support are central determinants of outcome rather than secondary technical issues. Anticoagulation during extracorporeal support remains particularly challenging because bleeding, thrombosis, platelet activation, inflammation, circuit complications, and suspected or confirmed heparin-induced thrombocytopenia often coexist. Individualized strategies are frequently required, while comparative evidence for heparin, bivalirudin, argatroban, and monitoring approaches remains limited [18,19,20].

## 5. Future Research Priorities

Several priorities should guide the next phase of research. First, cardiovascular phenotyping in the ICU should be standardized. Future studies should combine serial echocardiography, cardiac biomarkers, ECG patterns, vasopressor dose, fluid balance, lactate, inflammatory and endothelial biomarkers, shock severity staging, and clinical outcomes to distinguish septic cardiomyopathy, type 1 and type 2 myocardial infarction, Takotsubo syndrome, myocarditis, right ventricular failure, and non-ischemic myocardial injury.

Second, critically ill patients require dedicated trials rather than continued extrapolation from non-ICU cardiology populations. Evidence for antiplatelet therapy, anticoagulation, beta-blockers, antiarrhythmics, inotropes, heart failure therapies, and revascularization strategies is often generated in populations that exclude severe shock, multiorgan failure, or active bleeding risk. ICU-specific trial designs should include pragmatic endpoints, competing risks, and treatment interactions between organ support therapies.

Third, mechanical circulatory support research should move toward precision rather than expansion alone. Trials should test timing, unloading strategies, ECMO bundles, anticoagulation protocols, weaning criteria, bridge-to-decision pathways, neurological outcomes, quality of life, and cost-effectiveness. Right ventricular failure should also be studied as a distinct target in acute respiratory distress syndrome, pulmonary embolism, sepsis, post-transplant primary graft dysfunction, and VA-ECMO.

Fourth, survivorship should become a core endpoint in cardiovascular critical care research. ICU discharge or hospital survival is not enough. Future cardiovascular critical care studies should include functional recovery, cognitive outcomes, rehospitalization, rehabilitation needs, long-term heart failure trajectory, quality of life, and shared decision-making. This is particularly important in advanced heart failure, frailty, ECPR, and prolonged extracorporeal support.

## 6. Conclusions

The treatment of cardiovascular diseases in the critically ill will increasingly depend on moving from syndrome-based labels to biologically, hemodynamically, and functionally informed phenotypes. The contributions in this Special Issue highlight the breadth of this challenge, from advanced heart failure and frailty to fulminant myocarditis, ECPR, right ventricular support, and ECMO anticoagulation. Collectively, they reinforce the need for multidisciplinary collaboration across cardiovascular and intensive care teams. The next phase of progress should prioritize standardized definitions, multimodal monitoring, ICU-specific randomized trials, safer anticoagulation strategies, and patient-centered outcomes beyond survival.

## 7. Contributors to the Special Issue

The Guest Editor thanks all of the contributors to this Special Issue. The published articles addressed advanced heart failure risk stratification [21], frailty and resource utilization in heart failure [22], fulminant myocarditis supported with VA-ECMO [23], right ventricular primary graft dysfunction after heart transplantation [24], levosimendan use after extracorporeal cardiopulmonary resuscitation [25], and alternative anticoagulation for heparin-induced thrombocytopenia during ECMO [26]. Collectively, these contributions shaped the scope, academic coherence, and translational relevance of the issue.

## Data Availability

No new data were created or analyzed in this study.
